# Pseudotyping of HIV-1 with Human T-Lymphotropic Virus 1 (HTLV-1) Envelope Glycoprotein during HIV-1–HTLV-1 Coinfection Facilitates Direct HIV-1 Infection of Female Genital Epithelial Cells: Implications for Sexual Transmission of HIV-1

**DOI:** 10.1128/mSphere.00038-18

**Published:** 2018-04-04

**Authors:** Yuyang Tang, Alvin M. George, Oksana Petrechko, Franklin J. Nouvet, Stephanie D. Sweet, Yuetsu Tanaka, Brian S. Imbiakha, Guochun Jiang, Wei Gao, Kathryn Anastos, James E. K. Hildreth

**Affiliations:** a Department of Molecular and Cellular Biology, University of California—Davis, Davis, California, USA; b Department of Medical Microbiology and Immunology, University of California—Davis, Davis, California, USA; c Department of Obstetrics and Gynecology, University of California—Davis, Davis, California, USA; d Department of Immunology, Graduate School of Medicine, University of the Ryukyus, Okinawa, Japan; e Albert Einstein College of Medicine, Montefiore Medical Center, Bronx, New York, USA; f Department of Internal Medicine, Meharry Medical College, Nashville, Tennessee, USA; Icahn School of Medicine at Mount Sinai

**Keywords:** envelope glycoprotein, epithelial cells, human T-cell leukemia virus, human immunodeficiency virus, primary T-cells, pseudotype, retroviruses, sexual transmission, virus tropism

## Abstract

Female genital epithelial cells cover the genital tract and provide the first line of protection against infection with sexually transmitted pathogenic viruses. These cells normally are impervious to HIV-1. We report that coinfection of cells by HIV-1 and another sexually transmitted virus, human T-lymphotropic virus 1 (HTLV-1), led to production of HIV-1 that had expanded cell tropism and was able to directly infect primary vaginal and cervical epithelial cells. HIV-1 infection of epithelial cells was blocked by neutralizing antibodies against the HTLV-1 envelope (Env) protein, indicating that the infection was mediated through HTLV-1 Env pseudotyping of HIV-1. Active replication of HIV-1 in epithelial cells was demonstrated by inhibition with anti-HIV-1 drugs. We demonstrated that HIV-1 derived from peripheral blood of HIV-1–HTLV-1-coinfected subjects could infect primary epithelial cells in an HTLV-1 Env-dependent manner. HIV-1 from subjects infected with HIV-1 alone was not able to infect epithelial cells. These results indicate that pseudotyping of HIV-1 with HTLV-1 Env can occur *in vivo*. Our data further reveal that active replication of both HTLV-1 and HIV-1 is required for production of pseudotyped HIV-1. Our findings indicate that pseudotyping of HIV-1 with HTLV-1 Env in coinfected cells enabled HIV-1 to directly infect nonpermissive female genital epithelial cells. This phenomenon may represent a risk factor for enhanced sexual transmission of HIV-1 in regions where virus coinfection is common.

**IMPORTANCE** Young women in certain regions of the world are at very high risk of acquiring HIV-1, and there is an urgent need to identify the factors that promote HIV-1 transmission. HIV-1 infection is frequently accompanied by infection with other pathogenic viruses. We demonstrate that coinfection of cells by HIV-1 and HTLV-1 can lead to production of HIV-1 pseudotyped with HTLV-1 Env that is able to directly infect female genital epithelial cells both *in vitro* and *ex vivo*. Given the function of these epithelial cells as genital mucosal barriers to pathogenic virus transmission, the ability of HIV-1 pseudotyped with HTLV-1 Env to directly infect female genital epithelial cells represents a possible factor for increased risk of sexual transmission of HIV-1. This mechanism could be especially impactful in settings such as Sub-Saharan Africa and South America, where HIV-1 and HTLV-1 are both highly prevalent.

## Abstract (precis)

Young women in certain regions of the world are at very high risk of acquiring HIV-1, and there is an urgent need to identify the factors that promote HIV-1 transmission. HIV-1 infection is frequently accompanied by infection with other pathogenic viruses. We demonstrate that coinfection of cells by HIV-1 and HTLV-1 can lead to production of HIV-1 pseudotyped with HTLV-1 Env that is able to directly infect female genital epithelial cells both *in vitro* and *ex vivo*. Given the function of these epithelial cells as genital mucosal barriers to pathogenic virus transmission, the ability of HIV-1 pseudotyped with HTLV-1 Env to directly infect female genital epithelial cells represents a possible factor for increased risk of sexual transmission of HIV-1. This mechanism could be especially impactful in settings such as Sub-Saharan Africa and South America, where HIV-1 and HTLV-1 are both highly prevalent.

## INTRODUCTION

Although considerable progress has been made in limiting the spread of HIV-1, the global AIDS pandemic continues to expand in some regions of the world. Heterosexual transmission of HIV-1 is the primary route of HIV-1 dissemination worldwide (1). Multilayered squamous epithelium in the vagina and single-layer columnar epithelium in the cervix represent mechanical barriers to invasion by viruses and other pathogens. A number of hypotheses have been proposed to explain sexual transmission of HIV-1 in women. However, the mechanisms of HIV-1 transmission through the epithelial barrier have yet to be determined definitively. Genital mucosal epithelial cells do not express the major HIV-1 receptor CD4 and are normally impervious to infection with the virus. Direct transepithelial transcytosis of HIV-1 and transmigration of HIV-1-infected dendritic cells are the two major mechanisms that have been suggested to contribute to HIV-1 penetration of the vaginal mucosal epithelial layer (2, 3). The risk of HIV-1 transmission during sexual intercourse has been estimated to be quite low (1 in 1,000 to 1 in 200 encounters) (4–6). Therefore, under normal circumstances, it appears that the natural mechanical barrier represented by epithelial cells works effectively to prevent sexual transmission of HIV-1. However, in some regions of the world, such as Sub-Saharan Africa, HIV-1 transmission appears to occur at a very high frequency, especially in young women (7, 8). There is an urgent need to identify the factors that drive HIV-1 sexual transmission in this population group. A number of factors can substantially increase the risk of HIV-1 transmission, including ulceration, inflammation, and preexisting sexually transmitted infections (1, 5, 6). Sexual transmission is also impacted by the number of sex partners and the type of sex engaged in. In this study, we provide evidence that pseudotyping of HIV-1 with Env proteins from other sexually transmitted viruses could be a factor that contributes to the high frequency of infections in young women in Sub-Saharan Africa.

HIV-1 infection is frequently accompanied by coinfection with other viruses. It well documented that HIV-1 has the ability to incorporate other virus envelope glycoproteins during assembly, a phenomenon known as pseudotyping (9). This allows HIV-1 to expand its cellular tropism and enables it to infect non-CD4-expressing cells. In a recent proof-of-concept study, we demonstrated that when HIV-1 coinfects T cells with the *Gammaretrovirus* xenotropic murine leukemia virus-related virus, progeny HIV-1 particles are produced that are capable of infecting female genital epithelial cells (10). In the present study, we investigated whether coinfection of HIV-1 with another human-pathogenic retrovirus, human T-lymphotropic virus 1 (HTLV-1), can result in the production of HIV-1 particles pseudotyped with HTLV-1 Env protein and whether the pseudotyped HIV-1 can directly infect primary female genital epithelial cells.

Dual infection with HIV-1 and HTLV-1 has been reported in Sub-Saharan Africa, South America, and Caribbean countries and in high-risk patient groups (commercial sex workers and intravenous drug users) in Europe (11–13). The prevalence of coinfection with HTLV-1 and HIV-1 or HIV-2 has been reported to be >10% in several regions (14–25). Similar to HIV-1, HTLV-1 is transmitted via sexual contact, breastfeeding, blood transfusion, and the use of intravenous drugs. It has been suggested that HTLV-1 transmission from males to females is much more efficient than from females to males (26–28). In contrast to the tropism of HIV-1, which is limited to CD4^+^ immune cells, the tropism of HTLV-1 is broad and includes epithelial cells, as well as hematopoietic cells (29–33). Because the tropisms of HTLV-1 and HIV-1 overlap (i.e., CD4^+^ T cells) and they use distinct receptors, these viruses can coinfect the same cells and coinfection could facilitate acquisition of the HTLV-1 Env protein by HIV-1, rendering the latter virus capable of infecting non-CD4-expressing cells. Evidence that coinfection with HTLV-1 in T cell lines can broaden HIV-1 cellular tropism and enable it to infect non-CD4-expressing cells such as B cells and CD8^+^ T cells has been reported (34–38). However, whether pseudotyping of HIV-1 during coinfection with HTLV-1 could occur in primary T cells and *in vivo* has not been explored. Thus, the potential impact of pseudotyping of HIV-1 with HTLV-1 Env on sexual HIV-1 transmission is unknown.

Given the high prevalence of HIV-1–HTLV-1 dual infection globally and the ability of HIV-1 to incorporate HTLV-1 Env, it is likely that pseudotyping of HIV-1 by HTLV-1 Env could occur *in vivo*. The fact that pseudotyping allows HIV-1 to infect normally nonpermissive cells, especially cells in mucosal surfaces that serve as barriers against viral infection (such as epithelial cells in the genital tract), raises concern regarding HIV-1 transmission and pathogenesis in the setting of HTLV-1 coinfection. Here, we demonstrate pseudotyping of HIV-1 with HTLV-1 Env during coinfection both *in vitro* and *ex vivo*. Our data reveal that HTLV-1 Env-pseudotyped HIV-1 can be produced by primary CD4^+^ T cells coinfected with HIV-1 and HTLV-1 and that the pseudotyped HIV-1 is able to directly infect primary vaginal and cervical epithelial cells. We further demonstrate that HIV-1 derived from peripheral blood mononuclear cells (PBMCs) of HIV-1–HTLV-1-coinfected patients had broad cell tropism and was able to infect epithelial cells. Our finding of direct infection of primary genital epithelial cells by HLTV-1-pseudotyped HIV-1 establishes a possible mechanism for enhanced sexual transmission of HIV-1 in women in regions where both HIV-1 and HTLV-1 are highly prevalent.

## RESULTS

### Primary CD4^+^ T cells coinfected with HIV-1 and HTLV-1 transmit HIV-1 to primary female genital tract epithelial cells.

**FIG 1:**
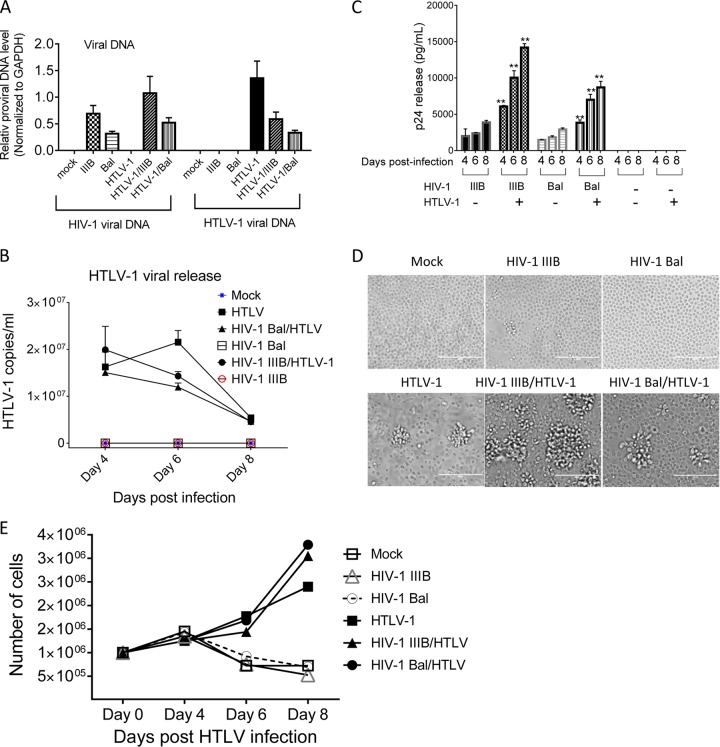
Coreplication of HIV-1 and HTLV-1 in primary CD4^+^ T cells. Primary CD4^+^ T lymphocytes were infected with HIV-1 IIIB or Bal and/or HTLV-1 as indicated. (A) HIV-1 IIIB or Bal and HTLV-1 DNA in infected cells at day 4 (HIV-1) or 5 (HTLV-1) postexposure was quantified by qPCR. (B) HTLV-1 production in culture supernatant at days 4, 6, and 8 postinfection was determined by qRT-PCR. (C) HIV-1 release at days 4, 6, and 8 postinfection was quantified by p24 ELISA. **, *P* < 0.001 (in comparison with data obtained with cells infected with HIV-1 alone). (D) Representative images show that exposure to HTLV-1 resulted in morphological changes in primary CD4^+^ T lymphocytes. Images were taken at day 5 after HTLV-1 exposure. (E) HTLV-1 exposure stimulated primary CD4^+^ T lymphocyte proliferation. The data shown represent the mean of data from three to six independent experiments.

Primary CD4^+^ T lymphocytes are major target cells for both HIV-1 and HTLV-1, and cells coinfected with these two viruses have the potential to produce HIV-1 pseudotyped with HTLV-1 *in vivo*. This hypothesis was first examined by using *in vitro* cell models. Primary CD4^+^ T lymphocytes were infected with HTLV-1 (39, 40) along with HIV-1 (R5 [Bal] or X4 [IIIB]). Primary CD4^+^ T cells infected with HTLV-1 or HIV-1 Bal or IIIB alone and mock-infected cells were included as controls. Active infection of primary CD4^+^ T lymphocytes with both HIV-1 and HTLV-1 was confirmed by quantifying *de novo* viral DNA in the cells (Fig. 1A) and virus release into culture supernatants (Fig. 1B and C). Primary CD4^+^ T cells exposed to HTLV-1 exhibited the characteristics of transformed cells (Fig. 1D) with enhanced cell growth (Fig. 1E). Our observations are consistent with previous reports that HTLV-1 triggers T cell activation and uncontrolled CD4^+^ T cell clonal expansion (41). HIV-1 and HTLV-1 coreplication was also confirmed in a T cell line chronically infected with HTLV-1 (MT2) after exposure to HIV-1 (see Fig. S1A in the supplemental material) and in the PM1 T cell line after inoculation with both retroviruses (Fig. S1B) by flow cytometry after immunostaining with antibodies against HTLV-1 p19 and HIV-1 p24Gag. It is noteworthy that HIV-1–HTLV-1 coinfection appeared to enhance HIV-1 replication while suppressing HTLV-1 replication relative to that in monoinfected cells (Fig. 1A to C). Whether this phenomenon would occur *in vivo* and impact pseudotyping is unclear.

10.1128/mSphere.00038-18.1 FIG S1  HTLV-1-positive MT2 (A) and HTLV-1-naive PM1 (B) cells were infected with the virus indicated. Cells were stained with antibodies against HTLV-1 p19 or HIV-1 p24 Gag and analyzed by flow cytometry to determine HIV-1 and HTLV-1 infection. Download FIG S1, TIF file, 0.1 MB. Copyright © 2018 Tang et al. 2018 Tang et al. This content is distributed under the terms of the Creative Commons Attribution 4.0 International license.

We next evaluated whether HIV-1 produced from HTLV-1-coinfected primary CD4^+^ T cells was pseudotyped with HTLV-1 Env and could infect nonpermissive cells such as female lower genital tract epithelial cells (FLGTECs; vaginal [VAG] and cervical [CER]). As noted above, HTLV-1 has wide cell tropism and is able to infect epithelial cells (30, 31). FLGTECs were isolated from vaginal and endocervical biopsy tissues as previously described (10). The susceptibility of FLGTECs to cell-associated HTLV-1 infection was then confirmed. HTLV-1 carrier CD4^+^ T cells (MT2) were mitotically inactivated with mitomycin C, which rendered the HTLV-1 carrier cells incapable of proliferating but allowed *de novo* HTLV-1 infection with cell-cell transmission. FLGTECs were then cocultured with the treated HTLV-1 carrier cells to facilitate cell-to-cell HTLV-1 infection. HTLV-1 infection of VAG and CER cells, as well as HeLa cells (a cervical epithelial cell line), was confirmed by staining intracellular HTLV-1 core protein p19 (Fig. S2A) and by quantifying *de novo* HTLV-1 DNA in the infected cells (Fig. S2B) and viral RNA released into culture supernatants (Fig. S2C).

10.1128/mSphere.00038-18.2 FIG S2  HTLV-1 infects primary female lower genital epithelial cells. (A) Representative images show HTLV-1 infection of HeLa and primary epithelial cells derived from the CER and VAG of healthy donors by immunofluorescence staining with anti-HTLV-1 core p19 MAb after coculture with HTLV-1-infected MT2 cells. (B) HTLV-1 DNA in infected epithelial cells was determined by qPCR. (C) HTLV-1 release in culture supernatant of infected epithelial cells and mock-infected control epithelial cells was quantified by HTLV-1-specific qRT-PCR. The data represent the mean ± the standard deviation from three independent experiments. HTLV-1^+^, epithelial cells exposed to HTLV-1-producing MT2 cells; HTLV-1^−^, mock-infected epithelial cells. Download FIG S2, TIF file, 0.2 MB. Copyright © 2018 Tang et al. 2018 Tang et al. This content is distributed under the terms of the Creative Commons Attribution 4.0 International license.

**FIG 2:**
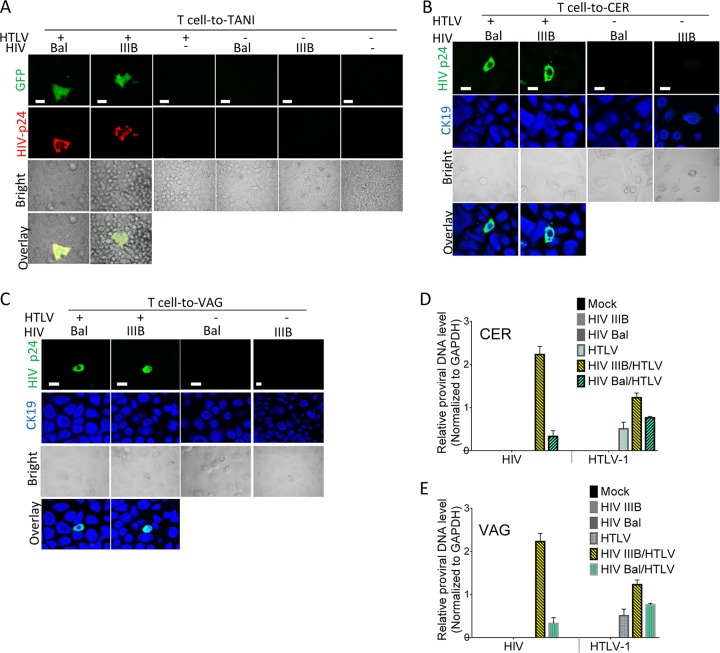
Infection of epithelial cells with HIV-1 from HIV-1–HTLV-1-coinfected CD4^+^ T cells. TANI reporter cells and primary epithelial cells isolated from VAG and CER biopsy tissues were infected by coculture with HIV-1 (Bal or IIIB)- and HTLV-1-coinfected (HTLV^+^), HIV-1-monoinfected (HTLV^−^), or mock-infected (HTLV^−^ HIV^−^) primary CD4^+^ T cells. The viruses used to infect donor primary CD4^+^ T cells are indicated in each panel. (A) HIV-1 infection of TANI reporter cells as indicated by GFP expression (green) and HIV-1 Gag expression (red) after immunostaining with anti-HIV-1 Gag antibody. (B, C) HIV-1 infection of primary CER (B) and VAG (C) epithelial cells. At day 5 postinfection, staining was performed with anti-HIV-1 Gag (green) and anti-CK19 (blue) antibodies, followed by confocal immunofluorescence microscopy analysis. The bright and merged views of the same field are shown (bottom). (D, E) DNA levels of both retroviruses in infected primary CER (D) and VAG (E) epithelial cells at day 5 postexposure were determined by qPCR. The data shown represent the mean ± the standard deviation from three independent experiments. Scale bars, 20 µm.

We next determined whether HIV-1 coproduced with HTLV-1 in CD4^+^ T cells was able to infect FLGTECs. Because HTLV-1 Env-mediated infection occurs primarily by cell-to-cell transmission (40, 42), this mode of infection was primarily used in the present study. Target FLGTECs or TANI reporter cells (HeLa cells expressing HIV-1 Tat-driven green fluorescent protein [GFP]) were cocultured with mitotically inactivated primary CD4^+^ T cells that were coinfected with HTLV-1 and HIV-1 IIIB or Bal. GFP and HIV-1 Gag expression in TANI cells confirmed infection and HIV-1 replication (Fig. 2A, two left panels). HIV-1 infection of CER and VAG cells was determined by dual intracellular staining for HIV-1 Gag and epithelial marker cytokeratin 19 (CK19) proteins after exposure to HIV-1 (IIIB or Bal)–HTLV-1-coinfected primary CD4^+^ T cells (Fig. 2B and C, two left panels) or T cell lines (Fig. S3A to C, left panels). The rate of HIV-1 infection of epithelial cells ranged from 1 to 10%. As expected, HIV-1-positive cells were not observed in epithelial cells exposed to T cells infected with HIV-1 alone or HTLV-1 alone or mock infected (Fig. 2A to C; Fig. S3A to C, right panels). Consistent with the immunostaining data, active HIV-1 infection was also confirmed by quantifying *de novo* HIV-1 DNA in infected primary CER (Fig. 2D) and VAG (Fig. 2E) epithelial cells exposed to HIV-1 IIIB–HTLV-1- or HIV-1 Bal–HTLV-1-coinfected primary CD4^+^ T cells and by quantifying HIV-1 release into culture supernatants from epithelial cells exposed to HIV-1 Bal–HTLV-1-coinfected PM1 cells (Fig. S3D). HIV-1 DNA and virus release were not observed in epithelial cells exposed to primary CD4^+^ T cells infected with HIV-1 or HTLV-1 alone or after exposure to mock-infected T cells (Fig. 2D and E; Fig. S2D). HTLV-1 replication in FLGTECs was confirmed by quantifying *de novo* HTLV-1 DNA in cells (Fig. 2D and E) or by HTLV-1 release (Fig. S3E) after exposure to HTLV-1-infected T cells or HIV-1–HTLV-1-coinfected T cells. Our data confirm that primary CD4^+^ T lymphocytes coinfected with HIV-1 and HTLV-1 were able to transmit HIV-1 to FLGTECs and initiate productive infection of these cells that are normally nonpermissive to HIV-1.

10.1128/mSphere.00038-18.3 FIG S3  HIV-1 infection of primary FLGTECs via coculture with HIV-1–HTLV-1-coinfected T cell lines. (A, B) Primary CER or VAG epithelial cells were cocultured with HIV-1 Bal- or IIIB-infected or mock-infected HTLV-1-positive MT2 cells as indicated. Epithelial cells were immunostained with antibodies against HIV Gag (green) or the epithelial cell marker CK19 (red). (C to E) Primary CER or VAG epithelial cells or HeLa cells were exposed to PM1 cells infected with the virus indicated. (C) Representative images showing HIV-1 infection of CER and VAG cells. Green fluorescence indicates HIV-1 Gag expression. Blue fluorescence indicates epithelial cell marker CK19 expression. Merged fields are shown in the bottom panels. (D, E) HIV-1 Bal (D) and HTLV-1 (E) release into culture supernatants of infected epithelial cells was quantified by HIV-1 p24 ELISA and HTLV-1 qRT-PCR, respectively. The data represent the mean ± the standard deviation of data from three independent experiments. Download FIG S3, TIF file, 0.3 MB. Copyright © 2018 Tang et al. 2018 Tang et al. This content is distributed under the terms of the Creative Commons Attribution 4.0 International license.

### Cell-free progeny HIV-1 particles produced by HIV-1–HTLV-1-coinfected CD4^+^ T cells infect primary VAG and CER epithelial cells.

**FIG 3:**
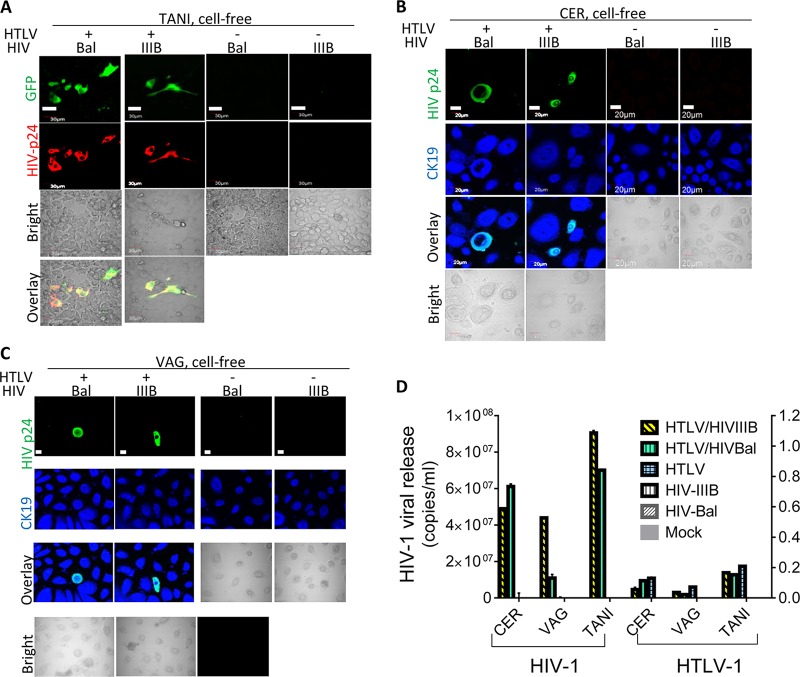
Infection of female genital epithelial cells by cell-free HIV-1 from HIV-1–HTLV-1-coinfected CD4^+^ T cells. TANI, CER, and VAG cells were infected with progeny virus from HIV-1 (IIIB or Bal)- and HTLV-1-coinfected primary CD4^+^ T cells or infected with progeny virus from control CD4^+^ T cells infected with HIV-1 or HTLV-1 alone or mock infected. Viruses used to infect donor primary CD4^+^ T cells are indicated in each panel. (A) TANI cells were stained with anti-HIV-1 Gag antibody and analyzed for GFP (green) and HIV-1 Gag (red) expression. Scale bars, 30 µm. (B, C) Primary CER and VAG epithelial cells were stained with anti-HIV-1 p24Gag (green) and anti-CK19 (blue) antibodies. Scale bars, 20 µm. (D) HIV-1 and HTLV-1 release into culture supernatants of CER and VAG primary epithelial cells, as well as TANI cells, was quantified by qRT-PCR at day 5 postinfection. The data shown represent the mean ± the standard deviation of data from three independent experiments.

HIV-1 is able to use both cell-associated and cell-free routes for infection. We next determined whether FLGTECs were permissive for cell-free HIV-1 coproduced with HTLV-1. HIV-1 released from primary CD4^+^ T lymphocytes or T cell lines coinfected with HTLV-1 and HIV-1 IIIB or Bal was added to TANI cells and FLGTECs. Equivalent amounts of HIV-1 from T cells infected with HIV-1 IIIB or Bal alone were included as controls. HIV-1 infection was detected only in epithelial cells exposed to HIV-1 that was coproduced with HTLV-1, as determined by GFP and Gag expression (TANI) or Gag expression (VAG, CER) (Fig. 3A to C, left two panels) and HIV-1 release (Fig. 3D). The frequency of infected epithelial cells was low (<1%) but highly reproducible. It is worth noting that the frequency of infected primary T cells after exposure to cell-free HIV-1 can be equally low (<1%). As expected, cell-free HIV-1 produced by T cells infected with HIV-1 alone did not infect epithelial cells nor was HIV-1 infection seen after exposure to cell-free HTLV-1 or supernatants from mock-infected T cells (Fig. 3A to C [right panels] and D). Quantitative reverse transcriptase PCR (qRT-PCR) analysis revealed low HTLV-1 production after exposure to cell-free HTLV-1 from HTLV-1-infected or HTLV-1–HIV-1-coinfected T cells (Fig. 3D), supporting earlier studies indicating that HTLV-1 virions are poorly infectious (43). These data show that cell-free HIV-1 coproduced with HTLV-1 in CD4^+^ T cells can directly infect primary genital epithelial cells. It is well established that infection with cell-free HTLV-1 occurs at low frequency (43, 44). Therefore, it is not surprising that the efficiency of infection with HTLV-1-pseudotyped HIV-1 was low since the infection is mediated by the envelope glycoprotein of HTLV-1.

### HIV-1 infection of female genital epithelial cells is inhibited by anti-HTLV-1 Env neutralizing antibodies and by anti-HIV-1 drugs.

**FIG 4:**
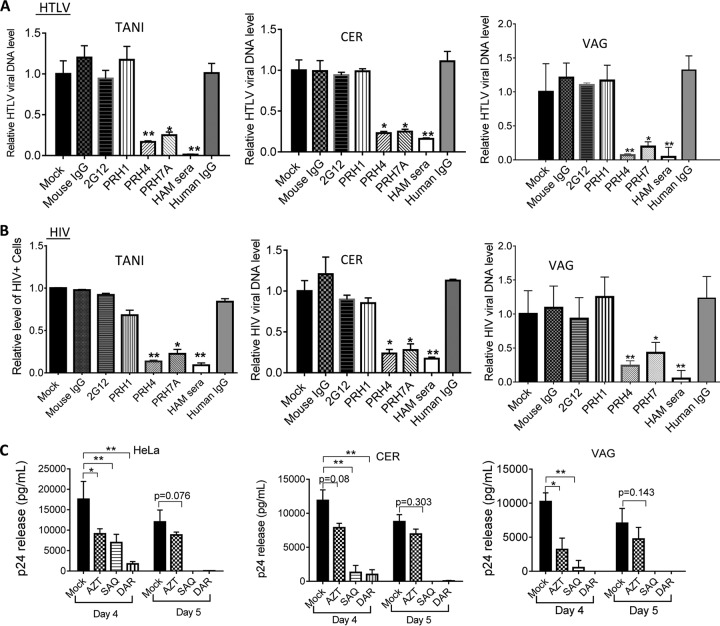
Infection with HTLV-1-pseudotyped HIV-1 was strongly inhibited by HTLV-1 neutralizing antibodies and HIV-1 inhibitor treatments. (A, B) TANI or primary VAG or CER epithelial cells were cocultured with HIV-1 IIIB–HTLV-1-coinfected T cells in the presence of antibodies as indicated. (A) *De novo* HTLV-1 DNA from infected TANI, CER, and VAG primary epithelial cells at day 5 postinfection was quantified by qPCR (normalized to the GAPDH internal control). (B) The effect of the antibodies on HIV-1 infection of epithelial cells was determined either by quantifying GFP-positive cells (TANI) or by quantifying the *de novo* level of HIV-1 DNA normalized to the GAPDH internal control (CER and VAG epithelial cells). PRH4 and PRH7A, neutralizing MAbs against HTLV-1 gp46; PRH1, HTLV-1 gp46 binding antibody without neutralizing activity; HAM serum, IgG from the serum of patients with HAM; 2G12, neutralizing MAb against HIV-1 gp120. Viral replication in mock-treated epithelial cells was set as 1.0. *, *P* < 0.05; **, *P* < 0.001. For PRH4 and PRH7A, data were compared to mouse IgG-treated samples. For HAM serum, data were compared to normal human IgG-treated samples. The data represent the mean ± the standard deviation of data from three independent experiments. The concentration used for PHR4, PRH7A, PRH1, and mouse IgG was 10 µg/ml. The concentration used for HAM serum IgG and normal human IgG was 100 µg/ml. The concentration used for 2G12 was 16 µg/ml. (C) HeLa cells and primary CER and VAG epithelial cells and were infected with HIV-1 by coculture with HIV-1 IIIB–HTLV-1-coinfected CD4^+^ T cells in the presence of AZT (10 µM), SAQ (0.4 µM), or DAR (0.5 µM). HIV-1 release into culture supernatants of epithelial cells was determined by p24 ELISA at days 4 and 5 postinfection. The data represent the mean ± the standard deviation of data from three independent experiments.

We showed that FLGTECs became susceptible to both cell-associated and cell-free HIV-1 that was coproduced with HTLV-1 in CD4^+^ T cells, implying that HIV-1 acquired HTLV-1 Env during the coinfection. To provide direct evidence that HIV-1 infection of epithelial cells was mediated by HTLV-1 Env, we next performed neutralization assays with antibodies against HTLV-1 Env (anti-HTLV-1 Env gp46 monoclonal antibodies [MAbs] PRH-7A and PRH-4 and IgG from patients with HTLV-1-associated myelopathy/tropical spastic paraparesis [HAM serum IgG]). The anti-HTLV-1 neutralizing activity of these antibodies has been previously demonstrated (45–47) and was confirmed in our study by measuring the HTLV-1 DNA in target FLGTECs and TANI cells after coculture with HTLV-1-infected cells in the presence of the antibodies (Fig. 4A). We next determined the neutralizing effects of PRH-7A, PRH-4, and HAM IgG on HIV-1 infection in cell-to-cell infection assays in which FLGTECs or TANI cells were cocultured with T cells coinfected with HIV-1 IIIB and HTLV-1. HIV-1 infection was assessed by quantifying the GFP-positive (TANI) cells by flow cytometry or by measuring *de novo* HIV-1 DNA (VAG and CER cells) by quantitative PCR (qPCR), respectively. HTLV-1 neutralizing antibodies PRH7A, PRH4, and HAM IgG all markedly inhibited HIV-1 infection of TANI cells and FLGTECs (Fig. 4B). Inhibition was not observed with nonneutralizing anti-HTLV-1 gp46 MAb PRH1 or with nonimmune mouse or human serum (Fig. 4B). The well-characterized HIV-1-neutralizing antibody 2G12 against gp120 also failed to block HIV-1 infection of epithelial cells (Fig. 4B). The ability of 2G12 to neutralize HIV-1 as previously reported (48) was confirmed with TZM-bl reporter cells (Fig. S4A). PRH7A, PRH4, or HAM IgG had no cross-neutralizing activity against HIV-1 infection, as determined in TZM-bl cells (Fig. S4B). These results confirm that infection of epithelial cells by HIV-1 produced from HTLV-1–HIV-1-coinfected T cells was mediated by HTLV Env.

10.1128/mSphere.00038-18.4 FIG S4  (A) Confirmation that MAb 2G12 neutralizes HIV-1. The neutralizing activity of MAb 2G12 against cell-associated HIV-1 was confirmed by exposing TZM-bl cells to PM1 cells infected with HIV-1 IIIB or Bal. The dilution of the antibody is indicated. Infection was assessed after 2 days by measuring luciferase activity as described in Materials and Methods. (B) HTLV-1 neutralizing antibodies did not inhibit HIV-1 infection. TZM-bl cells were exposed to cell-associated HIV-1 by coculture with PM1 cells infected with HIV-1 IIIB or Bal in the presence of the antibodies indicated. The antibody concentrations were the same as those described in the legend to Fig. 4. Download FIG S4, TIF file, 0.1 MB. Copyright © 2018 Tang et al. 2018 Tang et al. This content is distributed under the terms of the Creative Commons Attribution 4.0 International license.

Anti-HIV-1 drugs were used to further confirm active replication of HIV-1 in epithelial cells. Epithelial cells were exposed to HIV-1 IIIB–HTLV-1-coinfected T cells in the presence of the HIV-1 RT inhibitor zidovudine (AZT) or the protease inhibitors saquinavir (SAQ) and darunavir (DAR). SAQ and DAR dramatically decreased HIV-1 release at day 4 postinfection, and viral production fell below the detection limit at day 5 postinfection (Fig. 4C). Partial inhibition of HIV-1 replication by AZT treatment was also detected (Fig. 4C). As expected, the HIV-1 protease inhibitors SAQ and DAR and the RT inhibitor AZT showed no effect on HTLV-1 replication in FLGTECs, as determined by qRT-PCR assay of HTLV-1 RNA release into culture supernatants (Fig. S5A) and by immunostaining for HTLV-1 p19 (Fig. S5B). These results confirmed that active replication of HIV-1 was occurring in epithelial cells.

10.1128/mSphere.00038-18.5 FIG S5  HTLV-1 infection of FLGTECs was not affected by AZT, SAQ, or DAR treatment. (A) HeLa, VAG, and CER cells were exposed to HTLV-1-producing T cells (HTLV-1) or T cells coinfected with HTLV-1–HIV-1 IIIB in the presence of the HIV inhibitors indicated or mock treated. HTLV-1 release in culture supernatant was determined by HTLV-1-specific qRT-PCR at day 5 postinfection. The data represent the mean ± the standard deviation of data from three independent experiments. (B) HeLa, VAG, and CER cells were exposed to HTLV-1-infected CD4^+^ T cells in the presence of AZT. Epithelial cells were stained with anti-HTLV-1 p19 core antibody (red) and anti-CK19 antibody (blue). The overlay and bright views are shown in the right panels. The drug concentrations used were as follows: AZT, 10 µM; SAQ, 0.4 µM; DAR, 0.5 µM. Download FIG S5, TIF file, 0.2 MB. Copyright © 2018 Tang et al. 2018 Tang et al. This content is distributed under the terms of the Creative Commons Attribution 4.0 International license.

### HIV-1 produced by female genital epithelial cells infects naive CD4^+^ T cells and epithelial cells.

**FIG 5:**
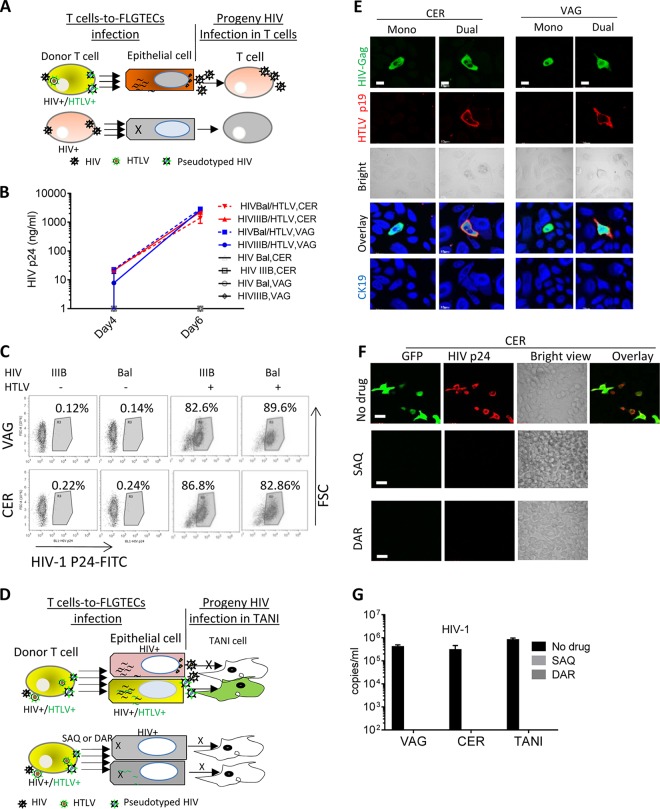
Genital epithelial cells infected with HIV-1 from HTLV-1-coinfected T cells efficiently transmit HIV-1 to susceptible T cells and also produce pseudotyped HIV-1. (A) Cartoon illustrating the system used to test the ability of primary epithelial cells HIV-1 infected through pseudotyping to produce infectious HIV-1. (B, C) The progeny virus from epithelial cells infected by coculture with infected T cells was added to naive PM1 cells. The viruses used to infect donor T cells and types of epithelial cells are as indicated. HIV-1 infection of PM1 cells was determined at days 4 and 6 postinfection by measuring HIV-1 release by p24 Gag ELISA (B) and at day 6 postexposure by quantifying HIV-1 p24 Gag-positive cells by flow cytometry (C). (D) Cartoon illustrating the system used to test whether HIV-1–HTLV-1-coinfected donor T cells can transmit both HIV-1 and HTLV-1 to epithelial cells, resulting in subsequent rounds of epithelial cell infection with pseudotyped HIV-1. SAQ and DAR should inhibit pseudotyped HIV-1 infection of epithelial cells and subsequent pseudotyped HIV-1 production. (E) HIV-1–HTLV-1-coinfected T cells transmitted both HIV-1 and HTLV-1 to epithelial cells. CER and VAG epithelial cells were infected by coculture with HIV-1 IIIB–HTLV-1-infected T cells and stained at day 5 postinfection for HIV-1 Gag (green), HTLV-1 p19 (red), and CK19. Mono, HIV-1 monoinfection; dual, HIV-1–HTLV-1 coinfection. Scale bars, 20 µm. (F, G) Epithelial cells were cocultured with HIV-1 IIIB–HTLV-1-coinfected T cells with or without the drugs indicated. Progeny HIV-1 from epithelial cells was added to TANI cells. (F) Images showing HIV-1 infection of TANI cells after exposure to progeny virus from infected CER cells as determined by analyzing GFP (green) and HIV-1 Gag expression (red). Scale bars, 20 µm. (G) HIV-1 release in culture supernatants from infected TANI cells at day 5 postexposure determined by qRT-PCR. The data represent the mean ± the standard deviation from three independent experiments.

The studies described above demonstrated that coinfection of HIV-1 with HTLV-1 resulted in the production of HTLV-1 Env-pseudotyped HIV-1 that was able to directly infect female genital epithelial cells. We hypothesized that the spread of infection from pseudotyped HIV-1-infected genital epithelial cells to intraepithelial T cells would be much more efficient than virus transmission from epithelial cells exposed to T cells infected with HIV-1 alone since the latter epithelial cells would not be productively infected with HIV-1 (Fig. 5A). To test this hypothesis, CD4^+^ T cells (PM1 cells) were exposed to culture supernatants from epithelial cells that had been infected with HIV-1 by coculture with T cells coinfected with HIV-1 and HTLV-1. As expected, HIV-1 infection of PM1 cells was detected after exposure to supernatants from FLGTECs cocultured with HIV-1–HTLV-1-coinfected T cells (Fig. 5B and C); no HIV-1 infection was detected in PM1 cells exposed to supernatants from FLGTECs cocultured with T cells infected with HIV-1 IIIB or Bal alone (Fig. 5B and C). These data revealed that epithelial cells infected with HTLV-1 Env-pseudotyped HIV-1 produced infectious HIV-1 capable of infecting permissive normal host cells.

Because both pseudotyped HIV-1 and HTLV-1 can infect epithelial cells, coreplication of HIV-1 and HTLV-1 in FLGTECs is possible and these coinfected epithelial cells should be able to release pseudotyped HIV-1 capable of infecting neighboring naive epithelial cells (Fig. 5D). To test this concept, we first confirmed HIV-1 and HTLV-1 coinfection of epithelial cells cocultured with HIV-1–HTLV-1-coinfected producer T cells. Triple immunostaining for HIV-1 Gag, HTLV-1 p19, and the epithelial cell marker CK19 revealed HIV-1–HTLV-1-coinfected epithelial cells (Dual), as well as epithelial cells infected with HIV-1 alone (Mono) (Fig. 5E). To determine whether the coinfected epithelial cells could release pseudotyped HIV-1 capable of infecting naive epithelial cells, progeny virus collected from VAG, CER, and HeLa cells cocultured with HIV-1–HTLV-1-coinfected T cells was used to infect TANI reporter cells. HIV-1 infection of TANI cells was confirmed by HIV-1 Gag and GFP expression (Fig. 5F, top panels) and by HIV-1 release (Fig. 5G). The results showed that HIV-1 pseudotyped with HTLV-1 Env was produced by FLGTECs and HeLa cells cocultured with HIV-1–HTLV-1-coinfected cells. As expected, blocking pseudotyped HIV-1 infection of epithelial cells with the protease inhibitor SAQ or DAR inhibited the subsequent pseudotyped HIV-1 infection of TANI reporter cells (Fig. 5D, F [bottom panels], and G). Overall, these results suggest that pseudotyping with HTLV-1 Env enables HIV-1 to directly infect FLGTECs. Moreover, since both HIV-1 and HTLV-1 can infect the same epithelial cells under these conditions, multiple rounds of HIV-1 infection of epithelial cells is possible through pseudotyping, further facilitating the spread of HIV-1 to intraepithelial CD4^+^ T cells, macrophages, or dendritic cells. This observation suggests that even though infection of epithelial cells by pseudotyped HIV-1 may occur at low frequency, the likelihood of transmission of HIV-1 may be enhanced through local spread of the virus to neighboring epithelial cells.

### HIV-1 from HTLV-1-coinfected subjects infects nonpermissive epithelial cells.

We have successfully demonstrated that *in vitro* coinfection of primary CD4^+^ T cells with HIV-1 and HTLV-1 leads to production of HTLV-1 Env-pseudotyped HIV-1 with expanded cell tropism including female genital epithelial cells. Given that both HIV-1 and HTLV-1 are highly prevalent in Sub-Saharan Africa, South America, and Caribbean countries and in high-risk patient groups (commercial sex workers and intravenous drug users) (11–13, 49–51), we next determined whether HIV-1 from HIV-1–HTLV-1-coinfected subjects was able to infect nonpermissive epithelial cells. The Women’s Interagency HIV Study (WIHS) recruited HIV-1–HTLV-1-coinfected subjects for a historical cohort study (52). HTLV-1 testing was done during the study enrollment period (from October 1994 through November 1995). Subjects visited WIHS clinical sites semiannually over a long period. Blood and cervicovaginal lavage (CVL) specimens from each visit were stored in the WIHS central repository. On the basis of availability, we selected specimens from the person visits of seven HIV-1–HTLV-1-coinfected subjects with the greatest plasma HIV loads (18 person visits in total, 2 to 4 visits/subject) (group 1). Specimens from 18 HIV-1-positive, HTLV-1-negative subjects recruited in the same cohort study with matching HIV-1 load and visit time criteria were included as the control group (group 2). Characteristics of both participant groups are shown in Table 1.

**TABLE 1:** Summary characteristics of the HIV-1–HTLV-1 dually infected group and the HIV-1-monoinfected control group

Characteristic	HTLV-1–HIV-1 dual infection	HIV-1 monoinfection
No. of subjects	18^a^	18
Median age, yr (IQR)^g^	46 (12)	41 (10)
No. (%) with HIV risk^b^		
Heterosexual	3 (42.9)	6 (33.3)
IDU^h^	3 (42.9)	4 (22.2)
Other	1 (14.3)	8 (44.4)
Median plasma HIV-1 RNA load^n^ (IQR)	4.8 (1.05)	5.0 (0.6)
No. (%)^c^ with CVL HIV-1 RNA load^n^ of:		
>1.7	4 (22.2)	4 (22.2)
<1.7	2 (11.1)	3 (16.7)
ND^j^	4 (22.2)	5 (27.8)
NA^k^	8 (44.4)	6 (33.3)
No. (%) with HTLV-1 RNA in plasma^c^		
Detected	1 (5.6)	0 (0)
Not detected	7 (94.4)	18 (100)
No. (%) with positive HTLV-1 serostatus^b,d^	7 (100)	0 (0)
No. (%)^c^ with CVL HTLV-1 RNA		
Detected	4 (22.2)	0 (0)
Not detected	14 (77.8)	18 (100)
Median no. of CD4^+^ cells/µl (IQR)^c^	151 (206.5)	324.5 (317.5)
No. (%)^e^ with ART use	15 (83.3)	2 (11.1)
No. (%) with LGT^i^ infection		
Oncogenic HPV^*f,l*^	18 (100)	16 (88.9)
HSV^*b,m*^	18 (100)	16 (88.9)
Bacterial vaginosis^c^	2 (11.1)	2 (11.1)
Candida vaginitis^c^	1 (5.6)	2 (11.1)

a Eighteen visits by seven HIV-1–HTLV-1-coinfected subjects were selected, two to four visits per subject.

b Data from the enrollment.

c Data from the time of sampling.

d As determined by Telzak et al. (52).

e Antiretroviral therapy (ART) in the past 12 months.

f Data from the first few visits.

g IQR, interquartile range.

h IDU, intravenous drug use.

i LGT, lower genital tract.

j ND, not detected.

k NA, data not available.

l HPV, human papillomavirus.

m HSV, herpes simplex virus.

n Log number of copies per milliliter.

Active virus replication of both retroviruses is required to produce HTLV-1 Env-pseudotyped HIV-1. The active replication of both retroviruses *in vivo* was determined by monitoring plasma viral RNA levels at the time of sampling. The plasma HIV-1 loads of both groups 1 and 2 at selected visits ranged from 4 to 6 log copies/ml (Tables 1 to 3). The data shown were generated by the WIHS cohort study investigators. In contrast to HIV-1 RNA, HTLV-1 RNA was only detected in plasma from seven HIV-1–HTLV-1-coinfected subjects at 1 of 18 selected person visits (subject 2 at visit 3, Table 2), indicating *in vivo* active HTLV-1 replication in this subject at the time of sampling. The HTLV-1 RNA level in the plasma from the remaining person visits of HIV-1–HTLV-1-coinfected subjects was under the detection limit (Table 2). Our data are consistent with previous observations that HTLV-1 maintains a long latent status *in vivo* and plasma HTLV-1 viremia is often not detected (53, 54).

**TABLE 2:** Virologic and clinical characteristics of women with HIV-1–HTLV-1 coinfection^g^

Subject and visit no.	HIV-1 RNA load^a^ in:		HTLV-1 RNA load^a^ in:		CD4^+^ cell count^b^	ART^c^
	Plasma	CVL	Plasma	CVL		
S1						
3	5.2	ND^e^	ND	4.6	142	AZT
4	3.8	<1.7	ND	ND	160	AZT
**S2**						
**3**	**5.7**	**2.82**	**5.45**	**5.7**	**646**	
17	6.0	1.95	ND	ND	455	AZT, 3TC, ABC
S3						
1^d^	4.0	NA^f^	ND	ND	35	AZT, DDI
2	4.3	ND	ND	ND	21	AZT, DDI, D4T
S4						
1^d^	4.8	NA	ND	ND	85	AZT, DDI
2	5.0	NA	ND	ND	28	AZT, DDI
5	4.9	2.6	ND	ND	120	RTV, DDI, D4T
12	4.7	2.2	ND	ND	128	SQV, D4T, DLV
**S5**						
1^d^	4.0	NA	ND	ND	261	AZT
**7**	**5.3**	**NA**	**ND**	**4.6**	**181**	**AZT, 3TC**
10	5.0	<1.7	ND	ND	140	AZT, D4T, 3TC, NVP
**24**	**4.3**	**ND**	**ND**	**ND**	**415**	**SQV, LPV, 3TC, TDF**
S6						
1^d^	4.8	NA	ND	ND	195	AZT
3	5.6	NA	ND	ND	141	AZT
S7						
1^d^	4.2	NA	ND	ND	317	
2	4.15	ND	ND	4.47	321	

a Log number of copies per milliliter at the time of sampling.

b Number of cells per microliter at the time of sampling.

c ART received in the last 12 months. Abbreviations: ART, antiretroviral therapy; DDI, didanosine; D4T, stavudine; RTV, ritonavir; DLV, delavirdine; 3TC, lamivudine; LPV, lopinavir; NVP, nevirapine.

d The duration of ART at the time of sampling at visit 1 is 6 months.

e ND, not detected.

f NA, data not available.

g Underlining and boldface indicate PBMCs from the person-visits which carry HIV-1 with expanded cell tropism to infect epithelial cells (boldface, without *ex vivo* stimulations; underlining, with pep005+JQ1 stimulation).

**TABLE 3:** Virologic and clinical characteristics of women from the HIV-1^+^ HTLV-1^−^ control group

Control subject no.	Visit no.	HIV-1 RNA load^a^ in:		HTLV-1 RNA load^a^ in:		CD4^+^ cell count^b^	ART^c^
		Plasma	CVL	Plasma	CVL		
C1	23	4.9	2.3	ND^d^	ND	64	3TC, ABC, EFV
C2	15	4.9	<1.7	ND	ND	53	
C3	2	5.4	2.8	ND	ND	615	
C4	13	5.1	2.1	ND	ND	247	
C5	13	5.0	<1.7	ND	ND	339	
C6	14	5.8	NA^e^	ND	ND	12	
C7	11	5.5	ND	ND	ND	102	
C8	22	4.3	3.7	ND	ND	379	
C9	3	5.4	NA	ND	ND	724	
C10	15	5.0	ND	ND	ND	398	
C11	16	5.4	NA	ND	ND	91	
C12	2	5.0	NA	ND	ND	381	
C13	6	4.7	<1.7	ND	ND	644	
C14	9	5.0	NA	ND	ND	310	
C15	18	5.0	ND	ND	ND	343	D4T, 3TC, NVP
C16	3	4.7	ND	ND	ND	283	
C17	19	4.8	ND	ND	ND	300	
C18	15	4.8	NA	ND	ND	472	

a Log number of copies per milliliter at the time of sampling.

b Number of cells per microliter at the time of sampling.

c ART received in the last 12 months. Abbreviations: 3TC, lamivudine; ABC, abacavir; ART, antiretroviral therapy; D4T, stavudine; EFV, efavirenz; NVP, nevirapine.

d ND, not detected.

e NA, data not available.

Despite the difficulties in detecting plasma HTLV-1 RNA, it was detected in CVL samples obtained from four HIV-1–HTLV-1-coinfected subjects (subject 1 at visit 3, subject 2 at visit 3, subject 5 at visit 7, and subject 7 at visit 2) (Table 2). HIV-1 RNA was detected in CVL samples from some subjects in the HIV-1–HTLV-1-coinfected group (Table 2) and in those from subjects in the HIV-1-positive, HTLV-1-negative control group (Table 3). These data suggest potential enhanced and/or compartmental replication of HTLV-1 in the genital tract and possible coreplication of both retroviruses in this compartment. As we expected, no HTLV-1 RNA was detected in plasma and CVL specimens collected at the selected visits from the HIV-1-positive, HTLV-1-negative group (Table 3).

**FIG 6:**
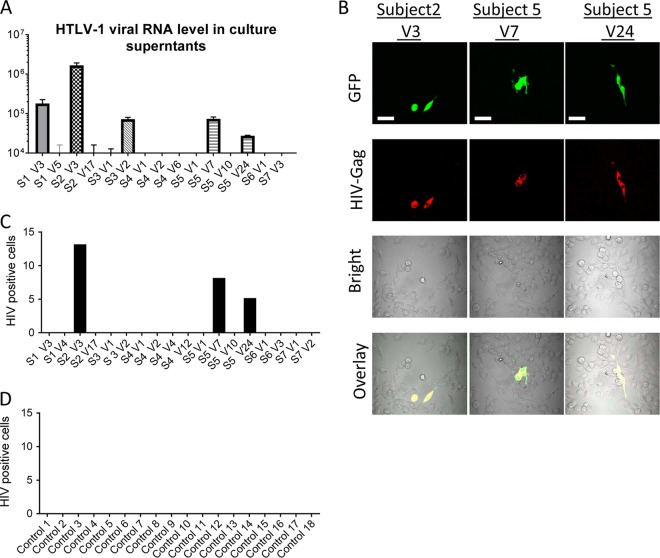
PBMC-associated HIV-1 from HIV-1–HTLV-1-coinfected patients infects epithelial cells. (A) HTLV-1 RNA released into culture supernatants of PBMCs from selected visits of HIV-1-infected, HTLV-1-seropositive subjects following culture for 2 days was determined by HTLV-1 specific qRT-PCR. S, subject; V, visit. (B to D) PBMCs from patients were used to infect epithelial reporter (TANI) cells by coculture. HIV-1 infection of TANI cells was determined by measuring GFP and HIV-1 Gag expression. (B) Representative images show that HIV-1 from PBMCs of HIV-1–HTLV-1-coinfected subject 2 at visit 3 and subject 5 at visits 7 and 24 of HIV-1–HTLV-1-coinfected groups were able to infect TANI reporter cells. Scale bars, 50 µm. (C, D) PBMCs from the indicated person visits of the HIV-1–HTLV-1 dually infected group (C) and from 18 HIV-1-monoinfected control subjects (D) were used to infected TANI cells. The number of HIV-1-infected TANI cells in a 1.5-cm^2^ area of the glass bottom dishes was quantified.

To determine whether HTLV-1 replicates *ex vivo* after short-term culture of PBMCs from HIV-1–HTLV-1-coinfected subjects, HTLV-1 RNA released into culture supernatants after 2 days of culture was measured. Relatively higher levels of HTLV-1 RNA were found in the culture supernatants of PBMCs from four HIV-1–HTLV-1-coinfected subjects at five visits (subject 1 at visit 3, subject 2 at visit 3, subject 3 at visit 2, and subject 5 at visits 7 and 24) (Fig. 6A), confirming *ex vivo* HTLV-1 replication in PBMCs from these subjects.

To assess the production of pseudotyped HIV-1 in cells from HIV-1–HTLV-1-coinfected subjects, we performed cell-associated infection assays by coculture of PBMCs from both groups 1 and 2 with TANI epithelial reporter cells. We observed that cell-associated HIV-1 from two HIV-1–HTLV-1-coinfected subjects (subject 2 at visit 3 and subject 5 at both visits 7 and 24) was able to infect the normally nonpermissive epithelial reporter cells, as determined by GFP and HIV-1 Gag expression (Fig. 6B and C). Notably, samples from these two subjects had detectable plasma HTLV-1 RNA and/or a high level of *ex vivo* HTLV-1 replication in PBMCs (Table 2; Fig. 6A). HIV-1 infection was not observed in the reporter cells that were exposed to PBMCs from the remaining HIV-1–HTLV-1-coinfected subjects or exposed to PBMCs from subjects infected with HIV-1 alone. These data indicate that pseudotyping of HIV-1 with HTLV-1 Env can occur both *in vivo* and *ex vivo* in cells of coinfected subjects and that active replication of both HTLV-1 and HIV-1 is required for production of pseudotyped HIV-1.

### Reactivation of latent HTLV-1 expression in PBMCs from HIV-1-infected and HTLV-1-seropositive subjects leads to production of pseudotyped HIV-1.

**FIG 7:**
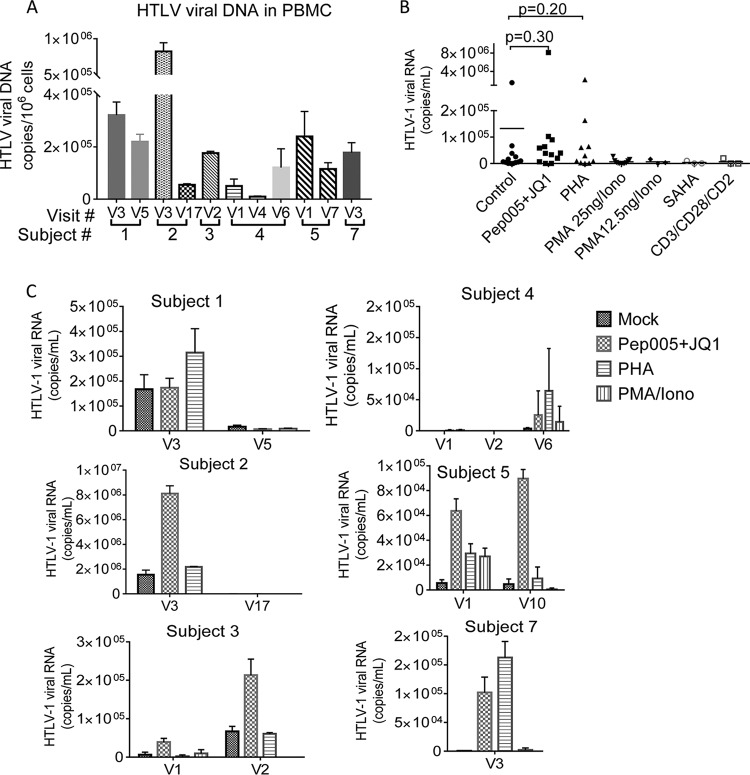
Reactivation of latent HTLV-1 in PBMCs from HIV-1–HTLV-1-coinfected subjects. (A) HTLV-1 DNA in PBMCs from selected person visits of the HIV-1–HTLV-1-coinfected group was quantified by qPCR. (B) PBMCs from the selected person visits of the HIV-1–HTLV-1-coinfected group were treated with the compounds indicated. HTLV-1 RNA in the culture supernatants was quantified at day 2 posttreatment. The final concentrations of the compounds used were as follows: PEP005, 20 nM; JQ1, 0.5 µM; PHA, 2 µg/ml; PMA 25 ng, PMA at 25 ng/ml plus ionomycin at 5 µM; PMA 12.5 ng, PMA at 12.5 ng/ml plus ionomycin at 5 µM; SAHA, 500 nM; CD3/CD28/CD2, 1× Stem Cell stock. (C) HTLV-1 RNA level in the supernatants of the PBMCs from each selected person visit of HIV-1–HTLV-1-coinfected donors after 2 days of stimulation with the compounds indicated. Data represent the mean ± the standard deviation from triplicate experiments. S, subject; V, visit.

Because HTLV-1 mostly maintains a long-term latent status *in vivo*, we next examined whether reactivation of latent HTLV-1 in PBMCs from HIV-1–HTLV-1-coinfected subjects resulted in the production of pseudotyped HIV-1. We first measured HTLV-1 DNA levels in PBMCs from coinfected subjects at selected visits. As expected, HTLV-1 DNA was detectable in PBMCs from all tested person visits of the HIV-1–HTLV-1-coinfected group (Fig. 7A). We next examined whether reactivation of latent HTLV-1 infection of PBMCs from HIV–HTLV-1-coinfected subjects would result in *de novo* or enhanced production of pseudotyped HIV-1. A recent study reported that PEP005-induced NF-κB signaling in combination with JQ1-induced p-TEFb activation could effectively reactivate latent HIV-1 (55). Since these two signal pathways also play important roles in HTLV-1 activation (56–58), we assessed the ability of this combination to reactivate HTLV-1 replication in PBMCs from HTLV-1–HIV-1-coinfected subjects. We also probed HTLV-1 reactivation with the standard activation stimulus phytohemagglutinin (PHA) or phorbol 12-myristate 13-acetate (PMA) plus ionomycin (PMA-iono) or with other published compounds. As shown in Fig. 7B and C, both the PEP005-JQ1 combination and PHA potently reactivated HTLV-1, as determined by qRT-PCR to quantify the HTLV-1 RNA released into culture supernatants at day 2 following stimulation. HTLV-1 reactivation was less effective with PMA-iono and other compounds (Fig. 7B and C).

**FIG 8:**
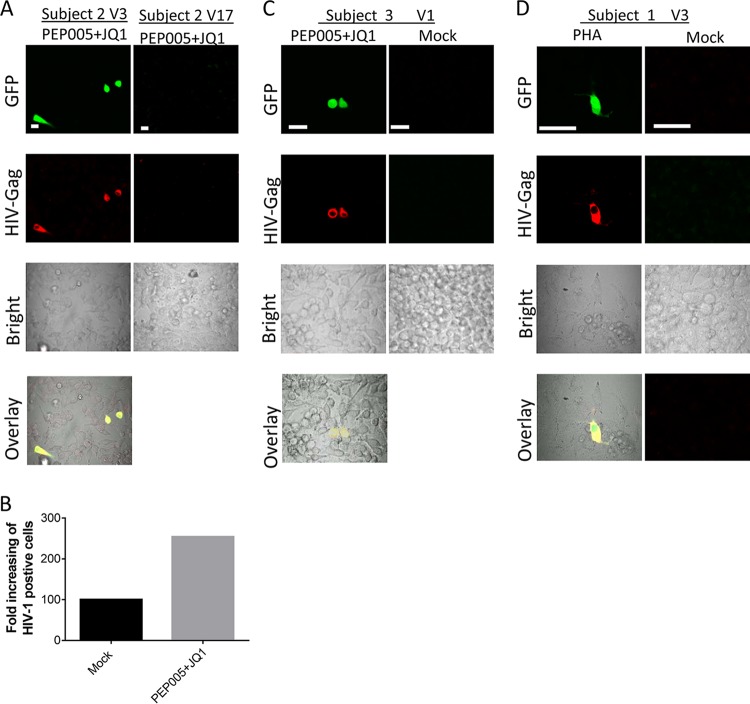
PBMCs from HIV-1-infected patients with latent HTLV-1 infection transmitted HIV-1 to epithelial reporter cells after reactivation of HTLV-1. PBMCs from HIV-1–HTLV-1-coinfected patients were treated with the compounds indicated for 2 days and then cocultured with TANI reporter cells. HIV-1 infection of TANI cells was determined by measuring GFP (green) and HIV-1 Gag (red) expression. (A) Representative images showing HIV-1 infection of TANI cells after coculture with PEP005- and JQ1-treated PBMCs from HIV-1–HTLV-1 dually infected subject 2 at visits 3 and 17. (B) Enhanced HIV-1 infection of TANI cells after stimulation of HTLV-1 replication in PBMCs from a HIV-1–HTLV-1-coinfected individual. TANI cells were cocultured with mock-treated or with PEP005- and JQ1-treated PBMCs from subject 2 at visit 3. The number of HIV-1-infected TANI cells in a 1.5-cm^2^ area of the glass bottom dish was quantified. HIV-1-positive TANI cells cocultured with mock-treated PBMCs were set as 100. (C, D) Representative images of HIV-1 infection of TANI cells after coculture with PBMCs from HIV-1–HTLV-1-coinfected subject 3 at visit 1 (C) and subject 1 at visit 3 (D) treated with the compound indicated or mock treated. Scale bars, 20 µm.

Subject 2 at visit 3 had extremely high levels of HTLV-1 DNA (Fig. 7A) and detectable plasma HTLV-1 RNA (Table 2). We have shown that unstimulated PBMCs from this subject at visit 3 transmitted HIV-1 to epithelial cells (Fig. 6B and C). PEP005-JQ1 treatment further increased HTLV-1 replication in PBMCs from this person visit (Fig. 7C) and resulted in enhanced HIV-1 transmission to TANI reporter cells (Fig. 8A and B). PBMCs from visit 17 of the same subject was not able to transmit HIV-1 to epithelial cells despite PEP005-JQ1 treatment or other *ex vivo* stimulation protocols (Fig. 8A), likely as a result of the low level of HTLV-1 DNA in PBMCs from that visit (Fig. 7A). We further observed that PBMCs from two subjects (subject 3 at visit 1 and subject 1 at visit 3) were able to transmit HIV-1 to epithelial reporter cells after the reactivation of latent HTLV-1 with PEP005-JQ1 or PHA (Fig. 8C and D). HIV-1 infection of epithelial cells was not detected after exposure to PBMCs from the remaining HIV-1–HTLV-1-coinfected subjects (subjects 4, 6, and 7), even when the same *ex vivo* stimulation procedures for HTLV-1 reactivation were applied. HIV-1 infection of the epithelial reporter cells was also not observed after the exposure of reporter cells to PBMCs from control subjects infected with HIV-1 alone, regardless of whether they were subjected to activation stimuli or not (data not shown).

Overall, we have shown that cell-associated HIV-1 derived from four of seven subjects coinfected with HIV-1 and HTLV-1 displayed expanded cell tropism and was able to infect normally nonpermissive epithelial cells. Among the four subjects, PBMCs from two subjects that showed *in vivo* and/or relatively high levels of *ex vivo* HTLV-1 replication during culture were able to transmit HIV-1 to epithelial cells without any *ex vivo* stimulation. PBMCs from the other two subjects were also able to transmit HIV-1 to epithelial cells after reactivation of latent HTLV-1 *ex vivo*. Our data highlight that when active replication of both HIV-1 and HTLV-1 occurs in primary T cells *in vivo* and *ex vivo*, pseudotyping of HIV-1 can and does occur, resulting in expanded HIV-1 tropism and infection of epithelial cells.

## DISCUSSION

In this study, we have shown that coreplication of HIV-1 and HTLV-1 results in HIV-1 pseudotyped with HTLV-1 Env both *in vitro* and *ex vivo*. This observation held true when these two retroviruses replicated in primary CD4^+^ T lymphocytes, as well as CD4^+^ T cell lines. Although published studies show that HIV-1–HTLV-1 coinfection of T cell lines can generate HIV-1 virions with expanded cell tropism that can infect non-CD4-expressing cell such as B cells and CD8^+^ T cells (35–38), these studies, which date back 30 years, were largely discontinued. Furthermore, how these observations related to HIV-1 transmission and pathogenesis was not explored. The data from our study show, for the first time, that primary VAG and CER epithelial cells are infected with pseudotyped HIV-1 released from HIV-1–HTLV-1-coinfected cells. We confirmed that HTLV-1 Env glycoprotein is required for HIV-1 infection of epithelial cells by blocking the infection with neutralizing antibodies against HTLV-1 Env. Infection was not blocked by neutralizing antibodies against HIV-1 gp120. Active replication of HIV-1 in epithelial cells was confirmed by inhibition of infection with anti-HIV drugs. Our data further revealed that productive infection of female genital epithelial cells resulted in release of pseudotyped HIV-1, which led to efficient HIV-1 transmission to susceptible CD4-positive target cells. In contrast to this, female genital epithelial cells exposed to T cells infected with HIV-1 alone failed to transmit HIV-1 to susceptible target cells. Therefore, our *in vitro* results support the concept that the direct infection of female genital epithelial cells by pseudotyped HIV-1 represents a highly efficient mechanism by which the virus can penetrate the epithelial cell barrier to transmission.

Our observation that cell-associated HIV-1 from HIV-1–HTLV-1-coinfected subjects was able to directly infect epithelial cells provides the first evidence to support pseudotyping of HIV-1 by another pathogenic virus *in vivo*. HTLV-1 RNA, as a marker of active viral replication, is not often detected in plasma from patients because of the latent status of HTLV-1 infection (53). Of seven HIV-1–HTLV-1-coinfected subjects from the WIHS cohort, one had high plasma HTLV-1 levels. Notably, cell-associated HIV-1 from this subject directly infected epithelial cells, strongly supporting the occurrence of pseudotyping of HIV-1 by HTLV-1 *in vivo* in coinfected subjects. We further observed that PBMCs from HIV-1–HTLV-1-coinfected subjects showing HTLV-1 replication *ex vivo* after reactivation of HTLV-1 replication were also able to transmit HIV-1 to epithelial cells. These results indicate that HIV-1 pseudotyping can potentially occur whenever there is active replication by both viruses in coinfected cells.

HTLV-1 was present in genital secretions but not in peripheral blood from three HIV-1–HTLV-1-coinfected subjects. This observation points to potentially higher levels of replication of HTLV-1 in the genital tract than in other peripheral compartments. Further studies are needed to confirm this intriguing observation. Since our *ex vivo* data indicate that pseudotyped HIV-1 can be present in the peripheral blood of HIV-1–HTLV-1-coinfected patients, it is reasonable to propose that pseudotyped HIV-1 particles and/or coinfected T lymphocytes could be present in genital secretions as well. Pseudotyped HIV-1 in the genital tract could result from either translocation from peripheral blood or local production by HIV-1–HTLV-1-coinfected cells in genital tract compartments. This idea was supported by our data showing that coinfection of epithelial cells by pseudotyped HIV-1 and HTLV-1 resulted in new rounds of infection of epithelial cells by pseudotyped HIV-1, implying that multiple rounds of infection of epithelial cells may occur in the setting of HIV-1–HTLV-1 coinfection. This potentially greatly increases the chances that the virus would be passed on to intraepithelial T cells, macrophages, or dendritic cells. Clearly HTLV-1-pseudotyped HIV-1 in genital secretions could enhance the sexual transmission of HIV-1, given our data showing direct infection of primary genital epithelial cells by HTLV-1 Env-pseudotyped HIV-1. Our results indicate that studies to understand cofactors driving coreplication of HIV-1 and HTLV-1 in genital compartments may be warranted, as are studies to determine whether the presence of pseudotyped HIV-1 in genital secretions is associated with significantly enhanced sexual transmission of HIV-1. In this study, we used female genital epithelial cells as targets for pseudotyped HIV-1. Our results may also have implications for female-to-male transmission of HIV-1, given the relative difficulty of transmission in this setting. This is particularly of interest since we obtained evidence that pseudotyping of HIV-1 with HTLV-1 occurs *in vivo* in coinfected women.

In summary, the present study shows that HIV-1–HTLV-1 coinfection results in pseudotyping of HIV-1 and expanded tropism of the virus to include primary female genital epithelial cells. This phenomenon provides a potential mechanism that facilitates vaginal transmission of HIV-1 and may be a contributing factor in the high prevalence of HIV-1 in settings such as Sub-Saharan Africa (59). HTLV-1 screening is not routine in HIV-1 patients and HIV-1–HTLV-1 coinfection likely occurs frequently but remains largely undiagnosed (11, 14). The ability of HTLV-1 Env-pseudotyped HIV-1 to infect primary female genital epithelial cells may, in part, explain the extremely high HIV-1 incidence and prevalence of HIV-1 among young women in Sub-Saharan Africa (7, 8). The significance of *in vivo* pseudotyping could extend beyond sexual transmission of HIV-1 since it has been reported that coinfection with HTLV-1 may influence HIV-1 disease progression (41, 50, 51, 60–64). The expanded tropism of HIV-1 as a consequence of pseudotyping and the consequent infection of nonhematopoietic cells could possibly explain, at least in part, changes in disease progression in HIV-1–HTLV-1-coinfected subjects.

## MATERIALS AND METHODS

### Cell culture.

MT2 (NIH AIDS Reagent Program), PM1 (NIH AIDS Reagent Program), and CEM × 174 (ATCC) cells were maintained in RPMI 1640 supplemented with l-glutamine, 10 mM HEPES (pH 7.2), and 10% fetal bovine serum (FBS). HeLa (ATCC) and TZM-bl (NIH AIDS Reagent Program) cells were maintained in complete Dulbecco’s modified Eagle’s medium (DMEM; Life Technologies, Inc.) DMEM was supplemented with 2 mM l-glutamine, 10 mM HEPES (pH 7.2), and 10% FBS. The TANI reporter cell line was created by stable transfection of HeLa cells with the pZsGreen1-DR-LTR plasmid (65), followed by selection with 800 μg/ml G418 (Sigma-Aldrich) over 2 weeks of culture. Cells were then cloned by limiting dilution in 96-well plates. Clones derived from single cells were selected and validated by infection with vesicular stomatitis virus G (VSVG)-pseudotyped HIV-1. HIV-1 infection was determined by immunostaining with an anti-HIV-1 Gag antibody. A clone (designated TANI) with low background expression and high GFP expression upon HIV-1 infection was chosen for reporter assays and maintained in complete DMEM in the presence of 800 μg/ml G418.

### Primary cell isolation and culture.

Details of the isolation and characterization of columnar and squamous primary epithelial cells from endocervical and vaginal biopsy tissues were described before (10). Written informed consent was obtained from all subjects, and the procedure was performed under the guidelines of a protocol approved by the institutional review board of the University of California at Davis. Anonymous leukopaks or Trima filters of peripheral blood samples were provided by the UCLA Center for AIDS Research. PBMCs were purified on Ficoll gradients by standard techniques. We isolated CD4^+^ T cells from PBMCs with immunomagnetic beads (Stem Cell Technologies). The purity of the isolated cells was assessed by flow cytometry after isolation, and the purity of CD4^+^ cells was >90%. Primary T cells were cultured in RPMI (Life Technologies, Inc.) supplemented with 10% FBS (HyClone), 2 mM l-glutamine, 10 mM HEPES (pH 7.2), 100 μg/ml streptomycin, 100 U/ml penicillin, and 20 U/ml interleukin-2 (R&D Systems).

### Preparation of HIV-1 and HTLV-1 stocks.

HIV-1 IIIB and Bal were prepared from chronically infected Jurkat T cells (ATCC) and PM1 cells, respectively. VSVG-pseudotyped HTLV-1 was produced by transfection of MT2 cells with a VSVG plasmid in a Nucleofector device (Lonza). HTLV-1 was produced from MT2 cells. Virus purification was done as described previously (10). HIV-1 was quantified by p24_Gag_ enzyme-linked immunosorbent assay (ELISA). The HTLV-1 p19_Gag_ content was determined by anti-HTLV-1 p19 ELISA (ZeptoMetrix Corporation), and the number of viral genomes in the viral stock was determined by HTLV-1-specific qRT-PCR.

### HIV-1 and HTLV-1 infection of producer T cells.

For HTLV-1 infection of CEM × 174, PM1, and primary CD4^+^ T cells, cell-free viral infection was done by spin inoculating cells with 150 ng/ml HTLV-1 or HTLV-VSVG at 1,200 × *g* for 2 h. Cells were infected with 200 ng/ml HIV-1 IIIB or Bal simultaneously with HTLV-1 or 1 day after HTLV-1 exposure. Cells infected with either virus alone were included as controls. We infected MT2 cells (chronically infected with HTLV-1) with 100 ng/ml HIV-1 IIIB or Bal. The cells were washed thoroughly to remove input virus 1 day after virus infection. For cell-mediated HTLV-1 infection of CEM × 174 and PM1 cells, MT2 cells were mitotically inactivated by treatment with 100 μg/ml mitomycin C at 37°C for 1 h and washed. Treated MT2 cells and uninfected CEM × 174 or PM1 cells were cocultured at a 1:1 ratio for 2 days and then infected with HIV-1 as described above.

### Cell-free and cell-to-cell infection of epithelial cells.

HeLa, TANI, and primary VAG and CER epithelial cells were seeded into 35-mm glass bottom dishes or 6-, 12-, or 24-well glass bottom plates (MatTek Corporation), depending on the experimental setup. For cell-free viral infection, each well typically received 200 ng/ml HIV-1 from HIV-1–HTLV-1-coinfected or HIV-1-infected T cells. Equivalent amounts of HTLV-1 from HTLV-1-infected T cells, as well as equivalent volumes of supernatant from mock-infected T cells were included as controls. The input virus was then removed by washing the cells 1 day after virus inoculation. For cell-associated infection, HIV-1–HTLV-1-coinfected T cells and control monoinfected T cells were treated with 100 μg/ml mitomycin C at 37°C for 1 h and washed. The T cells and epithelial cells were then cocultured for 2 days at a ratio of 1:1. The nonadherent T cells were removed by rinsing the epithelial cell monolayers three times with phosphate-buffered saline (PBS), and then growth medium was added back to the epithelial cells. For both cell-free and cell-associated infections, the epithelial cells and supernatants were harvested at day 5 postinfection.

### Intracellular immunostaining and confocal microscopy analysis and flow cytometry analysis.

Infected adherent epithelial cells in glass bottom dishes or plates were fixed with 4% paraformaldehyde for 30 min, and intracellular immunostaining was performed as described previously (10). Primary antibodies against HIV-1 p24_ Gag_ (KC57-fluorescein isothiocyanate [FITC]) and HTLV-1 p19 were used to stain HIV-1- and HTLV-1-infected cells, respectively. For double staining of infected cells with the epithelial cell marker CK19 and HIV-1 Gag, the cells were first stained with anti-CK19 primary antibody (Sigma) and then incubated with Cy3-conjugated goat anti-mouse IgG. The cell monolayer was then incubated with FITC-conjugated anti-HIV-1 Gag antibody (KC57-FITC). For triple staining of epithelial cells for HIV-1 p24_Gag_, HTLV-1 p19, and CK19, the cells were stained first with anti-HTLV p19 and Cy3-conjugated goat anti-mouse IgG and then labeled with KC57-FITC, followed by DyLight 405-conjugated anti-CK19 antibodies sequentially. The stained cells were examined with an Olympus FV1000 laser scanning confocal microscope (Olympus, USA). Flow cytometry analysis was performed to measure HTLV-1 and HIV-1 infection of producer T cells. Antibodies against HTLV-1 p19 (EMD Millipore) and HIV-1 p24_Gag_ (KC57-FITC; Beckman Coulter, Inc.) were used for intracellular immunostaining of fixed, permeabilized, infected T cells. To quantify the infection of HeLa cells exposed to pseudotyped HIV-1, KC57-FITC intracellular immunostaining and follow-up flow cytometry analysis were performed. Pseudotyped HIV-1 infection of TANI cells was determined by quantifying GFP-expressing cells.

### Quantitation of virus release and viral DNA.

HIV-1 and HTLV-1 release into supernatants of infected cells was determined either by HIV-1 p24 ELISA as described before (66) and HTLV-1 p19 ELISA (ZeptoMetrix Corporation) or by qRT-PCR measurement of viral RNA. RNA in culture supernatants from infected cells was isolated with QIAamp Viral RNA kits (Qiagen) and subjected to one-step qRT-PCR analysis with the iTaq Universal Probes One-Step kit (Bio-Rad). The primer-probe set used for the HIV-1 Gag-specific sequence was as reported before (67) (forward primer, 5′ CATGTTTTCAGCATTATCAGAAGGA 3′; reverse primer, 5′ TGCTTGATGTCCCCCCACT 3′; probe, 6-carboxyfluorescein [FAM]–5′ CCACCCCACAAGATTTAAACACCATGCTAA 3′–6-carboxytetramethylrhodamine [TAMRA]). The primer-probe set for HTLV-1 Tax-specific sequence was derived from M. Naderi and is as follows (68): forward primer, 5′ TTATCGGCTCAGCTCTACAGTTC 3′; reverse primer, 5′ GTGATTGGCGGGGTAAGGAC 3′; probe, FAM-5′ CGACTCCCCTCCTTCCCCACCCAG 3′-TAMRA. HIV-1 and HTLV-1 standard curves were generated with linearized HIV-1 plasmid pNL4.3 or HTLV-1 Tax plasmid (68). To quantify the HTLV-1 and HIV-1 DNA load in the infected T cells or the epithelial cells, cellular DNA was extracted with the QIAamp DNA minikit (Qiagen). HIV-1 and HTLV-1 qPCR analysis was performed by using iTaq Universal Probes Supermix (Bio-Rad) with the primer pairs listed above. The primer pair used for the endogenous human glyceraldehyde-3-phosphate dehydrogenase (GAPDH) control was as reported before (69): forward, 5′ TTGTTGCCATCAATGACCC 3′; reverse, 5′ CTTCCCGTTCTCAGCCTTG 3′. Real-time PCR assays were performed with a CFX96 real-time system (Bio-Rad).

### Neutralization assay.

Anti-HTLV-1 glycoprotein gp46 neutralizing MAbs PRH-7A and PRH-4 and control nonneutralizing HTLV-1 gp46 MAb PRH1 were gifts provided by Steven Foung of Stanford University and Jeffrey Lee of the University of Toronto. Purified anti-HTLV-1 serum IgG from HAM patients were obtained from Yuetsu Tanaka of the University of the Ryukyus, Japan. Normal nonimmune human IgG, mouse IgG (Jackson ImmunoResearch), and neutralizing anti-HIV-1 gp120-binding MAb 2G12 (Polymun Scientific Immunbiologische Forschung GmbH) were included as controls. Mitomycin C-treated HIV-1–HTLV-1-coinfected T cells were preincubated with appropriate concentrations of antibodies as described previously (45, 47) (PPH1, PHR4, PHR7A, and mouse IgG at 10 μg/ml; HAM anti-HTLV-1 IgG and normal human IgG at 100 μg/ml; 2G12 at 16 μg/ml). The donor T cells preincubated with antibodies were then cocultured with epithelial cells for 2 days, and the nonadherent T cells were then removed by thorough washing with PBS. Medium containing antibodies at the specified concentrations was present in the culture for another 24 h and then replaced with fresh medium without antibodies. Culture supernatants and epithelial cells were harvested at day 5 after virus exposure. The effects of antibodies on both HTLV-1 and HIV-1 production and infection of epithelial cells were determined by qPCR or immunofluorescence assay as described above. The effect of antibodies on HIV-1 replication in TZM-bl was determined by luciferase assay (70).

### Anti-HIV-1 drug treatment.

Epithelial cells were plated and cultured overnight as described above. Donor T cells coinfected with HIV-1 and HTLV-1 were mitotically inactivated by treatment with 100 μg/ml mitomycin C at 37°C for 1 h. Both epithelial cells and donor T cells were then pretreated with the HIV-1 RT inhibitor AZT (10 μM) or the HIV-1 protease inhibitor SAQ (0.4 or 2 μM) or DAR (0.5 μM) for 1 h at 37°C. These drug concentrations completely block HIV-1 infection of T cells *in vitro*. All of the drugs listed above were obtained from the NIH AIDS Reagent Program. The treated T cells and epithelial cells were then cocultured for 2 days. Epithelial monolayers were then rinsed with PBS three times to remove nonadherent infected T cells. Growth medium containing the inhibitors at the concentrations indicated was then added to the cells, and the inhibitors were present throughout the culture period. Supernatants from epithelial cells were harvested at days 3, 4, and 5 postinfection to quantify virus. Infected epithelial cells were then harvested at day 5 postinfection and subjected to intracellular immunostaining of HTLV-1 and HIV-1 proteins.

### Infectivity of progeny HIV-1 released by epithelial cells.

Primary VAG and CER epithelial cells and HeLa cells were infected by coculture with HIV-1 IIIB–HTLV-1-coinfected T cells for 2 days in the absence or presence of HIV-1 inhibitors (SAQ at 0.4 μM, DAR at 0.5 μM). The virus-containing supernatants were harvested at day 5 postinfection, filtered through 0.45-μm filters, and stored in a −80°C freezer. For infection of naive PM1 cells, 1 × 10^6^ PM1 cells were spin inoculated (1,200 × *g* for 2 h) at room temperature with 300 μl of supernatant from epithelial cells cocultured with HIV-1 IIIB–HTLV-1-coinfected or HIV-1 IIIB-infected T cells. The cells were then cultured overnight at 37°C and washed twice with PBS to remove the input virus before culture in complete medium. The supernatants were harvested at days 4 and 6 to assay HIV-1 release. The cells were fixed, permeabilized, and immunostained with anti-HIV-1 Gag MAb (KC57-FITC) before analysis by flow cytometry. For progeny HIV-1 infection of TANI cells, 300 μl of supernatant from epithelial cells cocultured as described above with T cells were spin inoculated onto TANI cells. HIV-1 release into supernatants was determined, and GFP and Gag expression in the infected cells was assayed.

### Study participants.

The WIHS is a longitudinal, multisite, observational cohort study of HIV-1 infection among United States women (70, 71). Plasma, serum, PBMCs, and cervical-vaginal lavage specimens are collected by WIHS on a semiannual basis and deposited at −80°C. HTLV-1 serologic tests of the initial wave of participants (enrolled in 1993-1994) were completed with an HTLV-1 indirect immunofluorescent antibody (IFA) test and confirmed by HTLV-1 and HTLV-2 Western blot assays. A total of 13 HIV-1-seropositive women were found to also have serologic reactivity to HTLV-1 (reported by Telzak et al. [52]). The present study utilized specimens from two groups of women from the WIHS cohort. The first group consisted of HIV-1–HTLV-1 dually infected women; the WIHS repository had samples available from seven HIV-1- and HTLV-1-seropositive women. For these dually infected women, we chose samples with the highest number of HIV-1 RNA copies in plasma (assessed over 18 study visits) for our study. The second group consisted of HIV-1-monoinfected women or controls (negative HTLV-1 serologic test results); we selected HIV-1-monoinfected and control samples from women/visits that were matched to women/visits in the HIV-1–HTLV-1 dual-infection group for HIV-1 load and CD4^+^ cell count. Samples from 18 women with HIV-1 monoinfection were selected for the present study. Cryopreserved and viable PBMCs and plasma and CVL fluid samples were retrieved from the WIHS repository and used for this study. Demographic and clinical characteristics of participants, including plasma and CVL fluid HIV-1 load data, were provided by WIHS cohort investigators.

### Laboratory measurements of patient specimens.

HTLV-1 RNA was measured by qRT-PCR as described above. HTLV-1 DNA in PBMCs was assessed by HTLV-1-specific qPCR. For infection assays with donor PBMCs, the cryopreserved viable PBMCs were cultured for 2 to 4 days in the presence or absence of treatment with different compounds to induce or enhance HTLV-1 replication. The concentrations of the compounds used were adapted from a previous study (55). The compounds were purchased from the following sources: PEP005 and JQ1, Tocris Bioscience; PHA, PMA, and suberoylanilide hydroxamic acid (SAHA), Sigma-Aldrich; anti-CD3, -CD28, and -CD2, Stem Cell Technologies. The treated PBMCs were then cocultured with TANI reporter cells at a ratio of 10:1 or 5:1. Two days later, the PBMCs were removed by rinsing the epithelial cell monolayers three times with PBS. Complete culture medium was added, and the epithelial cells and supernatants were harvested at day 5 postinfection. HIV-1 infection analysis was then done by immunostaining of the infected epithelial cells and measurement of virus release into culture supernatants.

### Statistics.

All experiments were repeated a minimum of three times. Statistical analysis was performed with GraphPad Prism 5.0 (GraphPad Software, Inc., La Jolla, CA). Comparisons of treated and control groups were performed by unpaired *t*-test analysis.
