# Supplementary figures and images for: Pseudotyping of HIV-1 with Human T-Lymphotropic Virus 1 (HTLV-1) Envelope Glycoprotein during HIV-1–HTLV-1 Coinfection Facilitates Direct HIV-1 Infection of Female Genital Epithelial Cells: Implications for Sexual Transmission of HIV-1
Source: mSphere. 2018 Apr 4;3(2):e00038-18. doi: 10.1128/mSphere.00038-18 (PMC5885023; doi:10.1128/mSphere.00038-18)

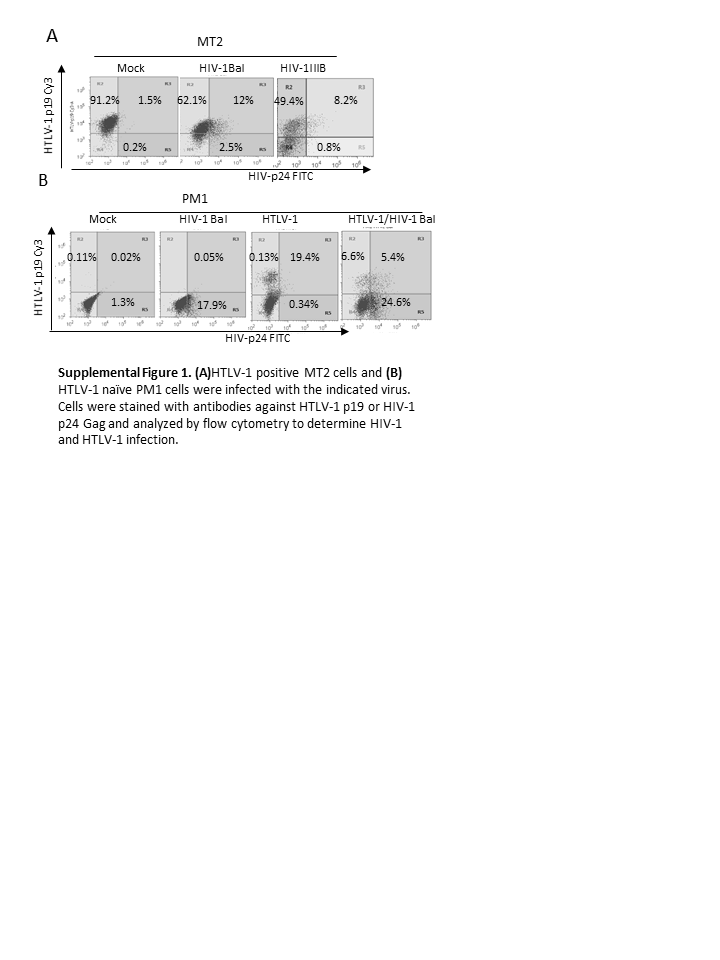

Supplement: FIG S1 [file sph002182507sf1.tif]

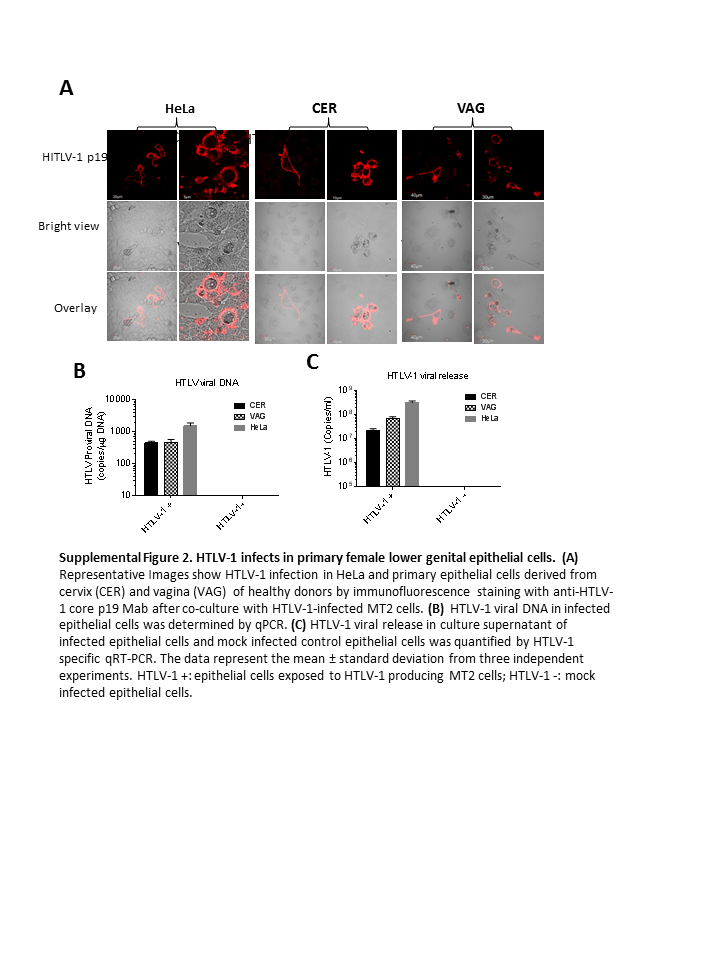

Supplement: FIG S2 [file sph002182507sf2.tif]

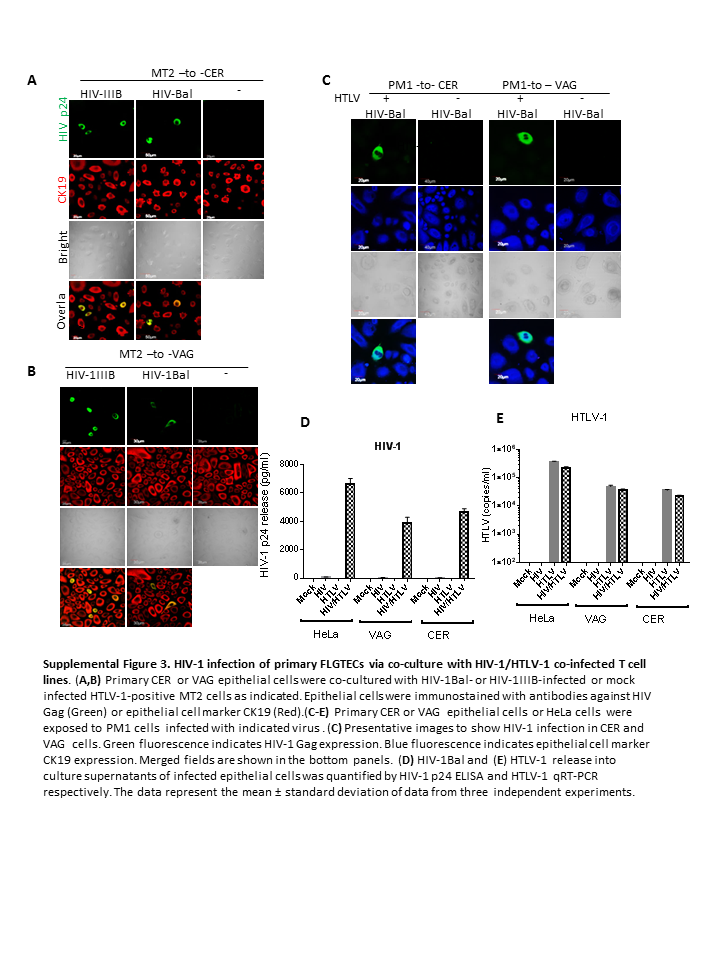

Supplement: FIG S3 [file sph002182507sf3.tif]

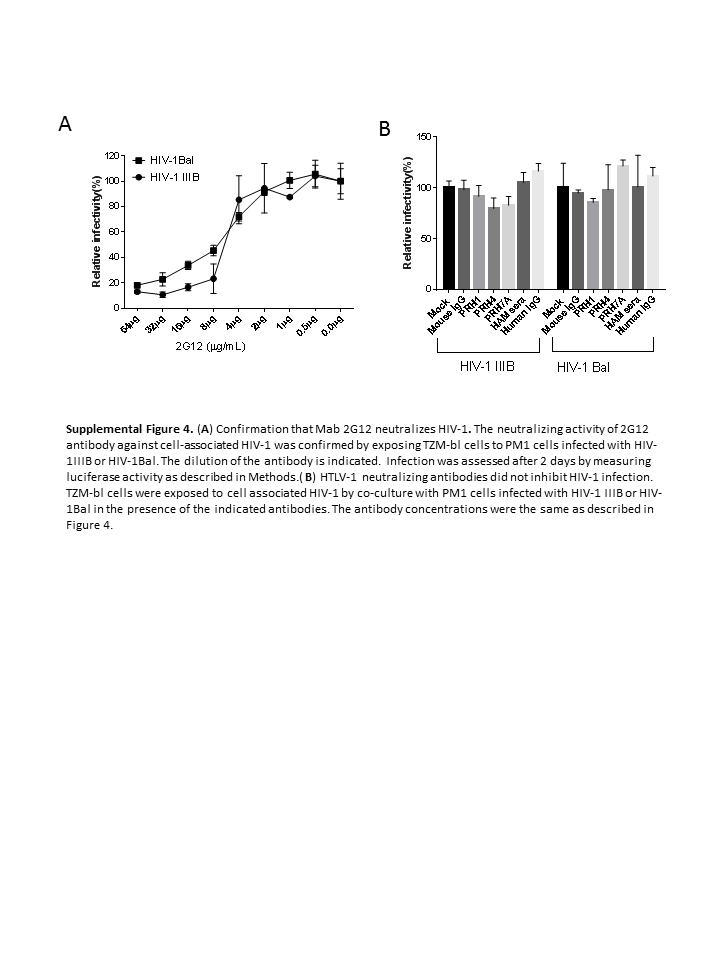

Supplement: FIG S4 [file sph002182507sf4.tif]

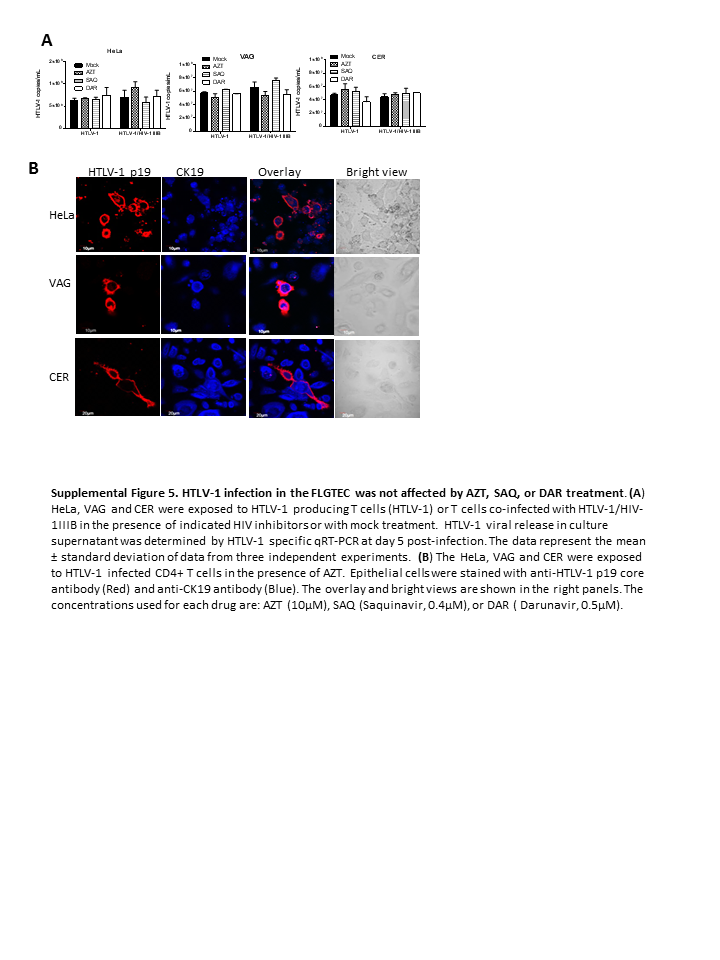

Supplement: FIG S5 [file sph002182507sf5.tif]
